# Introduction to “Transforming pregnancy since 1900”^☆^

**DOI:** 10.1016/j.shpsc.2014.07.001

**Published:** 2014-09

**Authors:** Salim Al-Gailani, Angela Davis

**Affiliations:** a Department of History and Philosophy of Science, University of Cambridge, United Kingdom; b Centre for the History of Medicine, University of Warwick, United Kingdom

Around 1900, few pregnant women in Western Europe or North America had any contact with a medical practitioner before going into labour. By the end the twentieth century, the hospitalisation of childbirth, the legalisation of abortion and a host of biomedical technologies from the Pill and IVF to obstetric ultrasound and prenatal diagnosis had dramatically extended the reach of science and medicine into human reproduction. This shift has a long and complex history which of course predates the introduction of twentieth-century innovations. Nevertheless, novel medical interventions such as ultrasound, many commentators assert, have transformed ‘the very experience of pregnancy’ (Petchesky, 1987). This special section originated in a workshop held in Cambridge in 2012. It stemmed from the observation that, despite a wealth of historical, sociological and anthropological writing on reproductive health and healthcare, we have a relatively insecure grasp of profound transformations in the science and management of pregnancy since the turn of the twentieth century. Existing historical research has been concerned primarily with the politics of childbirth and fertility control or framed within studies of the emergence of social policies focused on maternal and child welfare. By explicitly thematising continuity and change, the workshop aimed both to look beyond the most intensively studied topics and to contribute to ongoing reassessments of the ‘medicalisation’ of pregnancy as a historical process.

The decades around 1900 have long been understood as a formative period in the development of public health and social welfare services for women and children across the Western world. Widespread anxieties about national efficiency underpinned what John Pickstone has termed the ‘productionist’ political economy of early twentieth-century medicine: the assumed need to ensure healthy and numerous populations to supply the industrial labour force and military (Pickstone, 2000). Appeals for maternal and child health and welfare programmes emanated from across the political spectrum, often leading to intervention by both the voluntary sector and the state in the form of charity, medical care and social policy. As the ‘maternalist’ logic of these campaigns reconfigured reproduction as a national duty, the health of women during pregnancy and childbirth, as well as that of that of their newborn babies, took on unprecedented importance as a medical and political concern. The augmented power and coverage of both charitable and official public health services and the steady expansion of hospital-based maternity care in the early decades of the twentieth century, it has been argued, turned ‘pregnancy from a natural event into a medical problem’ (Seccombe, 1990, p. 181).

Since the late 1970s, ‘medicalisation’ has served as the dominant framework for the analysis of historical change in pregnancy and childbirth across the social sciences. Although it has since been defined in various ways, the theory of medicalisation can be traced to the emergence, during the 1970s, of a sociologically informed approach to the history of professions. Increasingly attending to issues of medical power and authority, sociologists interpreted the development of medicine, not only as a story of technical progress, but also in terms of the creation of a privileged and autonomous profession (Freidson, 1970). The medicalisation concept grew directly out of this broader interest in the growth of professional power as a function of social control and, in particular, as a way to theorise the extension of medical jurisdiction, authority and practices ever further into domains of everyday life. Most early sociological analyses of medicalisation assumed a direct and teleological relationship between the process of medical professionalisation and the growth of either a medical model of health or a medical regime allied to state power (Conrad, 1992, Zola, 1972). The term, often synonymous with the claim that society had become excessively medicalised, gained wider currency during the 1970s in the context of broader radical, libertarian and—most significantly with respect to pregnancy—feminist critiques of mainstream medicine.

Scholarship on the medicalisation of reproduction grew out of postwar demands for improvements in maternity care by patient consumer groups and especially the women’s health movement of the 1960s and 1970s. Particularly prominent in the United States, but increasingly influential internationally, the women’s health movement pushed debates over contraception, abortion and female autonomy in childbirth onto the political centre stage (Kline, 2010, Morgen, 2002). These concerns not only motivated the earliest second wave historical and social scientific studies of women’s healthcare, but also shaped much subsequent academic, as well as popular, writing on pregnancy and birth. Early second-wave studies, as Monica Green has put it, ‘articulated a historical past that conformed to the political present’ that activists were attempting to create: ‘where women could “once again” control their reproductive processes and be authorities in their own right on matters of their health’ (Green, 2008). In childbirth, this meant a ‘return’ to a golden age of ‘woman-centred’ and often home-based deliveries that had all but disappeared in the United States, Britain and many other industrialised countries.

The most influential writing on pregnancy from a historical perspective during the 1970s and early 1980s focused on struggles for the control of childbirth. These accounts presented late twentieth-century maternity care as the consequence of a historic power grab that had transformed obstetrics and gynecology into privileged and powerful professions at the expense of female midwives (Arney, 1982, Donegan, 1978, Donnison, 1977, Ehrenreich and English, 1973). This perspective emerged in a political context in which not only feminists but also non-feminist patient consumer organisations were campaigning for the redistribution of power between medical specialists and pregnant women, and for greater choice in childbirth (Oakley, 1984, pp. 236–249). Critics of maternity care—especially in the United States and Britain—argued that by redefining the ‘natural’ process of childbearing as inherently risky, obstetricians had deceived women into accepting hospital and medical interference as the rule for all births (Arms, 1975). Despite their different approaches these authors shared an understanding of the medicalisation of reproduction as a process that was, *a priori*, imposed upon women to their detriment; what British feminist sociologist Ann Oakley evocatively termed ‘the captured womb’ in her influential book of that title, published in 1984. Still one of the only historical studies to look beyond childbirth to consider the management of pregnancy in all its aspects, *The Captured Womb* explained the development of antenatal care and modern obstetrical interventions as strategies for the ‘social control of women’ by the medical profession on behalf of the state (1984, pp. 250–274).

Beginning in the 1980s, however, social historians of medicine, health and welfare—many taking up Michel Foucault’s refined notion of biopolitical power—challenged received accounts of medicalisation as the nefarious collaboration of experts and state authority imposed from above. In questioning earlier views of medicalisation as top-down and unidirectional, historians increasingly emphasised the importance of recovering patient demands and wider social contexts in order better to appreciate processes of change (Nye, 2003). This trend was particularly apparent in new social and cultural histories of midwifery, childbirth and early twentieth-century maternal and child welfare, which problematised social control models that left little room for women’s agency (Fildes et al., 1992, Lewis, 1980, Williams, 1997). In a groundbreaking book, American historian Judith Walzer Leavitt persuasively argued that transformations in childbirth, including the move to hospital, ‘directly reflected women’s needs at various points in history’ (Leavitt, 1986). According to the sociologist Jane Lewis, the author of several influential historical studies of gender and social policy, it is a ‘mistake to see women as passive recipients or victims of these changes’; instead ‘the process of medicalisation must be carefully differentiated’ (Lewis, 1990, p. 1). Using local case studies, historians also uncovered the tensions between national policies and the practice of maternity care in different regional settings, thus portraying the medicalisation of reproduction as a complex and uneven process (Davis, 2011, Marks, 1996, Nuttall, 2011). Less inclined to portray women as inert ‘victims’ of modern, hospital-based obstetrics, historians now more typically stress the role of ‘female collusion in the process, and its class dimensions and implications’ (Greenlees and Bryder, 2013, McCray Beier, 2004).

Feminist critiques of high technology obstetrics have also informed a second major strand of social scientific and historical writing on pregnancy. This concerns what Ilana Löwy, in her contribution to this special section, terms the ‘irresistible rise of the visible fetus’. Beginning in the 1980s feminist social scientists have analysed the ways in which ‘pro-life’ activism since the legalisation of abortion, especially although not only in the United States, has intersected with developments in medicine and technology to produce a burgeoning fascination with fetuses (Duden, 1993, Franklin, 1991, Morgan and Michaels, 1999, Petchesky, 1987). The increasingly pivotal role of obstetric ultrasound in maternity care since the 1970s and the growth of the sub-specialism of fetal medicine has reinforced feminist concern about the trend to reduce pregnant women to passive vessels of ‘unborn patients’ (Casper, 1998, Roberts, 2012, Taylor, 2008). In recent years, this enlargement of the fetus in medicine and wider culture has drawn fresh critique of the surveillance and moral regulation of pregnant women (Armstrong, 2003, Daniels, 1993, Lupton, 2013). Historians are beginning to document how and with what consequences women’s experiences of pregnancy came to revolve around a fetus (Buklijas and Hopwood, 2008, Dubow, 2011, Golden, 2005, Hanson, 2004, Nicolson and Fleming, 2013, Reagan, 2010) but, quite apart from assessing the impact of medical technology, there remains much to explore.

Recognising the need to recover the contingencies that shaped women’s experiences of pregnancy, recent histories have done much to complicate received accounts of the rise of obstetrics and the concomitant disempowerment of birthing women. But despite a wealth of research, there remains an overall tendency to view the history of pregnancy with respect to professional struggles over childbirth, especially between obstetricians and midwives, and in relation to state policy, particularly around the expansion of hospital-based maternity care and the legal status of abortion. Historians can expand upon the dominant frameworks by calling attention to the dynamic processes involved in the production of knowledge, practices and discourses around pregnancy among medical experts and the wider public. Contributions to this special section seek to build on existing work by taking into account the myriad actors and agendas implicated in changes in the management and experience of pregnancy across the whole twentieth century, and by assessing their consequences. Looking beyond obstetricians, midwives and maternity hospitals, the essays examine relations among pregnant women, less researched medical professionals—from biomedical researchers and laboratory technicians, to family doctors and genetic counsellors—, the broader healthcare industries and lay groups. Together they demonstrate the value of bringing into view the networks of individuals, institutions and technologies that have made and remade understandings of pregnancy since 1900.

Jesse Olszynko-Gryn’s essay explores the expansion of laboratory pregnancy testing as a routine practice in the early twentieth century. Despite considerable recent historical and social scientific interest in such postwar diagnostic technologies as obstetric ultrasound and amniocentesis, pregnancy testing has largely escaped attention. Olszynko-Gryn focuses on the Aschheim-Zondek reaction, generally recognised as the first reliable hormonal pregnancy test, institutionalised and used on a large-scale in Britain by a diagnostic laboratory in Edinburgh in the 1920s and 1930s. Explaining the success of the test in terms of the growth of the commercial laboratory services industry, he challenges the view that doctors promoted the Aschheim-Zondek reaction in order to extend the medical surveillance of normal pregnancy and assert the authority of obstetric knowledge over that of pregnant women. Olszynko-Gryn’s emphasis on the demand of predominantly medical ‘diagnostic consumers’ for a tool to detect malignant tumours and hormonal deficiencies believed to cause miscarriage invites us to attend closely to relations among users of technologies of pregnancy in order to understand their establishment and maintenance in clinical routine.

Miscarriage in general has been largely overlooked in the historical literature. Focusing on Britain in the first half of the twentieth century, Rosemary Elliot recovers the close connection between medical understandings of criminal abortion and spontaneous miscarriage. Aiming to open up wider questions about continuity and change in discourses of pregnancy loss, her essay explains why and with what consequences doctors began to identify spontaneous miscarriage as a field of medical expertise in the early decades of the twentieth century. Elliot stresses the need to historicise both medical and lay understandings of pregnancy loss, which have been shaped by changing attitudes to abortion, access to healthcare and medical technology. But her analysis also suggests continuities between early twentieth-century doctors’ and infant welfare reformers’ growing concerns about miscarriage as a public health problem and discourses around pregnancy loss, abortion and fetal rights in the present day.

Also attending to the significance of discourse, Angela Davis builds on a body of work that explains how approaches to and perceptions of pregnancy and birth in the twentieth century both defined and were defined by gender, and notably shifting idealisations of women’s identities as mothers (Davis, 2012, Plant, 2010). Historical metanarratives of twentieth-century maternity care often assume a simple dichotomy between ‘medical’ and ‘social’ views of childbearing. Davis challenges the medical-social binary by examining the interplay between narratives of pregnancy and birth and narratives of war in the accounts of maternity by women of the wartime generation. Drawing on oral history interviews conducted with British women who had experienced World War Two as children or adults and had their children either during the war or in the years soon after, her essay identifies the close association between maternity and military service during this time. Davis’s analysis encourages us to recognise how such narratives reflect the wider cultural context in which women gave birth.

Tatjana Buklijas identifies World War Two as a transformative period in clinical and scientific ideas about pregnancy and the fetus. Her essay relates how heightened interest in food and nutrition during and immediately after the war gave rise to a new concern with fetal growth and development, especially within biochemistry, physiology and agriculture. Through detailed engagement with the clinical and experimental work of the Cambridge nutrition scientists Robert McCance and Elsie Widdowson, Buklijas explains how low birth weight came to be seen as a sign of pathological pregnancy. Her account illuminates the significance of the wartime experience, not only in generating institutional, professional and social investments in prenatal nutrition, but also in the articulation of the concept of ‘critical periods’, used across several disciplines to describe the relationship between chronological time and the timing of developmental milestones. By tracing the reconceptualisation of pregnancy as a plastic, open state, Buklijas shows how McCance’s and Widdowson’s research set the scene for a resurgence of interest in the interplay between development and environment—particularly nutrition—in the late twentieth century.

The intersections between nutrition and reproductive science are also highlighted in Salim Al-Gailani’s analysis of preconceptional and prenatal vitamin supplementation in late twentieth-century Britain. Since the 1990s, a daily regimen of folic acid pills, understood to reduce the risk of having a baby with a neural tube defect, has become a routine part of the experience of pregnancy. The acceptance of folic acid as a ‘risk-reducing drug’ was also crucial to the development of a novel set of preventive and clinical practices concerned with women’s health before pregnancy, or ‘preconceptional care’. Al-Gailani traces the history of this innovation, but his larger concern is to show that public health policies to promote the consumption of folic acid by women of childbearing age are a symptom of a broader trend in which management of risk to the fetus has become the primary focus of medical intervention in pregnancy and childbirth. Focusing on clinical research into preconceptional vitamin supplementation and the controversies it stimulated, his account places transformations in the experience and management of pregnancy in relation to the politics of abortion and disability in the late twentieth century.

The emergence in the 1970s of the ‘prevention of disability’, and Down Syndrome in particular, as a political and public health concern is equally central to Ilana Löwy’s analysis of the development of prenatal diagnosis. Löwy describes the innovation of a new *dispositif*, a dynamic and evolving array of techniques and approaches that provide information about the fetus during pregnancy, in the postwar period. The result of a coming together of three medical innovations—amniocentesis, the study of human chromosomes and obstetrical ultrasound—with a social innovation, the decriminalisation of abortion, prenatal diagnosis has profoundly shaped the experience of pregnancy. In Löwy’s analysis, the transformation of prenatal diagnosis into a screening tool in many industrialised countries has been a crucial factor in the conceptualisation of pregnancy as a risky enterprise. Building on work by anthropologists Barbara Katz Rothman (1986) and Rayna Rapp (2000), her account emphasises that prenatal diagnosis and screening has compelled many women to view their pregnancies as a tentative state fraught with uncertainty. Löwy also offers the crucial insight that the ‘irresistible rise’ of the fetus as the focus of medical attention was, in the main, an invisible revolution; in spite of its profound consequences, the generalisation of prenatal diagnosis has largely escaped public scrutiny.

Also concerned with the permeation of risk discourse through all aspects of childbearing, sociologists Aryn Martin and Kelly Holloway revisit a transformative episode in the histories of pregnancy and drug regulation: the international medical tragedy precipitated by the widespread prescription of thalidomide to pregnant women as a sedative and antinauseant. Recognised in the early 1960s as the cause of birth defects on a massive and global scale, thalidomide is known to historians primarily as a media scandal and a pharmaceutical disaster. Martin and Holloway instead take the thalidomide tragedy as the starting point for a history of the concept of the ‘placental barrier’ and its twentieth-century trajectory. Drawing on evidence from specialist journals, obstetrics textbooks, and pregnancy advice manuals, their essay examines the cultural work performed by the maxim that thalidomide disabused both medical experts and the lay public of the notion that the placenta acts as a protective shield for the fetus. Critically assessing the historical accuracy of this claim, Martin and Holloway suggest that nostalgia for a barrier lost in fact tells us more about the emergence of an autonomous and agential fetus and dramatic changes in norms regulating women’s conduct during pregnancy in the late twentieth century.

The essays that follow draw on a range of sources to reflect on these manifold transformations from a variety of perspectives. However, the collection as a whole is far from comprehensive in its coverage, whether from a geographical, chronological or thematic perspective, and should be understood more as an invitation to further research than as a definitive statement. In including contributions by historians and sociologists working on the question in very different ways, we hope that our collection opens a number of avenues for further enquiry.
